# Administration of *Ligilactobacillus salivarius* CECT 30632 to elderly during the COVID-19 pandemic: Nasal and fecal metataxonomic analysis and fatty acid profiling

**DOI:** 10.3389/fmicb.2022.1052675

**Published:** 2022-12-16

**Authors:** Marta Mozota, Irma Castro, Natalia Gómez-Torres, Rebeca Arroyo, Isabel Gutiérrez-Díaz, Susana Delgado, Juan Miguel Rodríguez, Claudio Alba

**Affiliations:** ^1^Department of Nutrition and Food Science, Complutense University of Madrid, Madrid, Spain; ^2^Department of Microbiology and Biochemistry, Dairy Research Institute of Asturias (IPLA-CSIC), Villaviciosa, Spain

**Keywords:** probiotics, *Ligilactobacillus salivarius*, elderly, metataxonomics, fatty acid, nursing home, COVID-19

## Abstract

Elderly was the most affected population during the first COVID-19 and those living in nursing homes represented the most vulnerable group, with high mortality rates, until vaccines became available. In a previous article, we presented an open-label trial showing the beneficial effect of the strain *Ligilactobacillus salivarius* CECT 30632 (previously known as *L. salivarius* MP101) on the functional and nutritional status, and on the nasal and fecal inflammatory profiles of elderly residing in a nursing home highly affected by the pandemic. The objective of this *post-hoc* analysis was to elucidate if there were changes in the nasal and fecal bacteriomes of a subset of these patients as a result of the administration of the strain for 4 months and, also, its impact on their fecal fatty acids profiles. Culture-based methods showed that, while *L. salivarius* (species level) could not be detected in any of the fecal samples at day 0, *L. salivarius* CECT 30632 (strain level) was present in all the recruited people at day 120. Paradoxically, the increase in the *L. salivarius* counts was not reflected in changes in the metataxonomic analysis of the nasal and fecal samples or in changes in the fatty acid profiles in the fecal samples of the recruited people. Overall, our results indicate that *L. salivarius* CECT 30632 colonized, at least temporarily, the intestinal tract of the recruited elderly and may have contributed to improvements in their functional, nutritional, and immunological status, without changing the general structure of their nasal and fecal bacteriomes when assessed at the genus level. They also suggest the ability of low abundance bacteria to train immunity.

## Introduction

The coronavirus disease-2019 (COVID-19), caused by the Severe Acute Respiratory Syndrome Coronavirus 2 (SARS-CoV-2), has been the responsible for several millions of deaths around the world, being recognized by the WHO as a pandemic on 11 March 2020. Elderly was the most affected population during the first COVID-19 waves. In fact, the risk of a severe course, functional and nutritional complications, and mortality from COVID-19 was much higher among older people (≥ 65 years) than among younger people (Boccardi et al., 2020; Liu et al., 2020; Silva et al., 2020; Pizarro-Pennarolli et al., 2021; Palavras et al., 2022). Such a strong impact on elderly has been explained on the basis of an accumulation of risk factors, including multiple key comorbidities, such as, among others, weakened immune systems, hypertension, diabetes, cardiovascular disease, chronic kidney disease, and chronic respiratory disease (Araújo et al., 2021). Among the elderly, those living in nursing homes represented the most vulnerable group until vaccines became available (Aguilar-Palacio et al., 2022). The huge impact of the initial pandemic waves on this group has been linked to the pre-COVID-19 living conditions in long-term care facilities, which facilitated the spread of the virus among highly vulnerable people (Lai et al., 2020; Araújo et al., 2021).

Some studies have indicated an implication of the host microbiota in the individual susceptibility to COVID-19 and in the severity of the disease (Gu et al., 2020: Zuo et al., 2020; Albrich et al., 2022). People with an altered respiratory or gut microbiota would be at a higher risk of suffering a more severe infection, complications, and sequela because of their inability to develop correct immune responses (He et al., 2020a,b). Interestingly, aging has a negative impact on the composition of the gut microbiota (Claesson et al., 2011) and this aging-driven altered microbiota usually promotes inflammation (Guigoz et al., 2008).

In this context, the modulation of the respiratory tract and gut microbiotas and their associated immune responses may be a strategy to minimize the impact of COVID-19 and to foster a full recovery in vulnerable populations. In a previous article, we described the beneficial effect of a probiotic strain (*Ligilactobacillus salivarius* CECT 30632) on the functional and nutritional status, and on the nasal and fecal inflammatory profiles of elderly residing in nursing homes highly affected by the pandemic (Mozota et al., 2021). In this work, we present the results of the metataxonomic analysis performed with the nasal and fecal samples of a subset of these patients, before and after the administration of the strain for 4 months and, also, its impact on their fecal fatty acids profiles.

## Materials and methods

### Study design and participants

The general design of the trial (an open-label trial without a control arm) has already been published (Mozota et al., 2021). Briefly, the study was carried out in an elderly nursing home located in Moralzarzal (Madrid, Spain), and was designed to include all the residents as long as (a) informed consent was obtained from the participants or their legal representatives, (b) they were not fed by parenteral nutrition exclusively, and/or (c) they were not allergic to cow’s milk proteins (because the probiotic was delivered in a dairy food matrix). A total of 25 residents, aged 74–98, met these criteria and started the trial. Starting at day 0, the residents consumed daily a fermented dairy product (125 g; ~ 9.3 log10 CFU of *L. salivarius* CECT 30632 per product) for 4 months. Two samples (nasal wash and feces) were collected from each patient at recruitment (day 0) and at the end of the study (day 120). The nasal wash was obtained using a standardized protocol (Stewart et al., 2017). Aliquots of the samples were stored at −80°C until the analyzes were performed. This study was conducted according to the guidelines laid down in the Declaration of Helsinki and was approved by the Ethics Committee of the Hospital Clínico San Carlos (Madrid, Spain) (protocol: CEIC 20/263-E_COVID; date of approval: 01/04/2020, act 4.1/20).

In a previous work, the functional, cognitive and nutritional status of the patients was evaluated, according to standard procedures, before and after the administration of the probiotic strain, and their nasal and fecal inflammatory profiles were also measured (Mozota et al., 2021). A total of 22 out of the 25 recruited participants finished the trial. After the immunological analysis of their samples, and due to the low amount available of some them, we only kept aliquots of the nasal and fecal samples from a subset of 15 residents, which are the ones that have been analyzed in this study. The demographic and health-related data (age, gender, body mass index, SARS-CoV-2 status, comorbidities, and medication) that were recorded at recruitment and at the end of the study for this subset of residents are shown in Table 1; Supplementary Table S1, respectively. Their samples were submitted to culture-based and culture-independent analyzes and, additionally (in the case of the fecal samples), to the analysis of their fatty acids’ profiles, following the procedures described below.

**Table 1:** Demographic characteristics of the elderly population (*n* = 15).

	Mean (95% and CI) or *n* (%)
Age (years)	84.73 (75.87–93.60)
**Gender**	
Male	7 (46.67%)
Female	8 (53.33%)
**BMI (kg/m2)**	
Day 0	24.61 (20.64–28.57)
Day 120	24.47 (21.56–27.39)
**SARS-CoV-2 (positive PCR)**	
Day 0	11 (73%)
Day 120	0 (0%)

### Detection and quantification of *Ligilactobacillus salivarius* in the fecal samples by culture-dependent methods

Fecal samples collected during the trial were serially diluted and plated onto agar plates of MRS (Oxoid, Basingstoke, UK) supplemented with L-cysteine (2.5 g/l; MRS-Cys) for isolation of lactobacilli. MRS-Cys plates were incubated anaerobically (85% nitrogen, 10% hydrogen, 5% carbon dioxide) in an anaerobic workstation (DW Scientific, Shipley, UK) for up to 72 h at 37°C. After incubation, colonies were enumerated and at least one representative of each colony morphology was selected from the agar plates in order to calculate the *L. salivarius* count and the total *Lactobacillus* count. The latter parameter included all the new genera in which this genus was reclassified recently (Zheng et al., 2020). The isolates were identified by Matrix Assisted Laser Desorption Ionization-Time of Flight (MALDI-TOF) mass spectrometry (Bruker GmbH, Bremen, Germany) and 16S rDNA sequencing (Mediano et al., 2017). The isolates identified as *L. salivarius* were genotyped by RAPD profiling as described (Ruiz-Barba et al., 2005) to assess if they shared the same profile that *L. salivarius* CECT 30632.

### DNA extraction from the nasal and fecal samples

Two different protocols were performed depending on the type of biological sample. For nasal samples (1 g), DNA was extracted following the protocol described by Pérez et al. (2019). This protocol includes a first centrifugation at 13,000 rpm for 10 min at 4°C, an incubation with lysozyme (5 mg/ml), mutanolysin (25,000 U/ml), and lysostaphin (4,000 U/ml), a mechanical lysis using the FastPrep BIO 101 instrument (Thermo Scientific, Waltham, MA) and an incubation with proteinase K (250 μg/ml) at 56°C for 30 min. Then, the DNA was extracted using the QIAamp DNA Stool Kit (Qiagen, Hilden, Germany), following the instructions of the manufacturer. The protocol described by Lackey et al. (2019) was used for the fecal samples (1 g). In all cases, DNA was eluted in 20 μl of nuclease-free water and its concentration was estimated with a ND-1000 UV spectrophotometer (Nano Drop Technologies, Wilmington, DE, USA).

### Detection and quantification of *Ligilactobacillus salivarius* in the fecal samples by real-time quantitative PCR (qPCR) assays

Quantification of *L. salivarius* DNA in the fecal samples of the residents was carried out using the procedure described by Harrow et al. (2007). The DNA concentration of all samples was adjusted to 5 ng/μL. A commercial real-time PCR thermocycler (CFX96™, Bio-Rad Laboratories, Hercules, CA, USA) was used for all experiments. Standard curves using 1∶10 DNA dilutions (ranging from 2 ng to 0.2 pg) from *L. salivarius* CECT5713 were used to calculate the concentrations of the unknown bacterial genomic targets. Threshold cycle (Ct) values between 14.92 and 21.15 were obtained for this range of bacterial DNA (*R*^2^ ≥ 0.991). The Ct values measured for DNA extracted from two strains belonging to two non-target species (*Lactiplantibacillus plantarum* MP02 and *L. reuteri* MP07; our own collection) were ≥ 39.36 ± 0.57. These control strains were selected because they are closely related, from a taxonomical point of view, to *L. salivarius* (Salvetti et al., 2018). All samples and standards were run in triplicate.

### Metataxonomic analysis

The 16S rRNA gene amplification and sequencing, targeting the V3-V4 hypervariable regions of the 16S rRNA gene, was performed in the MiSeq 300PE system of Illumina (Illumina Inc., San Diego, CA, United States) at the facilities of Parque Científico de Madrid (Tres Cantos, Spain) with the universal primers S-D-Bact-0341-b-S-17 (ACACTGACGACATGGTTCTACACCTACGGGNGGCWGCAG) and S-D-Bact-0785-a-A-21 (TACGGTAGCAGAGACTTGGTCTGACTACHVGGGTATCTAATCC), as previously described (Klindworth et al., 2013; Aparicio et al., 2020). The pooled, purified, and barcoded DNA amplicons were sequenced using the Illumina MiSeq pair-end protocol (Illumina Inc.).

Demultiplexing preprocessing analysis of the V3-V4 amplicons was conducted using MiSeq Reporter analysis software (version 2.6.2.3), according to the manufacturer’s guidelines. After the demultiplexing step, the metataxonomic analyzes were conducted with QIIME 2 2022.2 (Bolyen et al., 2019). Denoising and ASVs (Amplicon sequence variants) selection were performed with DADA2 (Callahan et al., 2016). The forward reads were truncated at position 290 by trimming the last 10 nucleotides, while the reverse ones were truncated at the 249 nucleotides by trimming the last 8 nucleotides, in order to discard nucleotides in positions for which median quality was Q20 or below.

Taxonomy was assigned to ASVs with the q2-feature-classifier (Bokulich et al., 2018) by using a classify-sklearn naïve Bayes taxonomy classifier against the SILVA 138.1 reference database (Quast et al., 2013). Subsequent bioinformatic analysis was conducted using R version 3.5.1 (R Core Team, 2021).^1^ The decontam package version 1.2.1 (Davis et al., 2018) was used to identify, visualize, and remove contaminating DNA with one negative extraction control. A table of amplicon sequence variants (ASVs) counts per sample was generated, and bacterial taxa abundances were normalized with the total sum scaling normalization method, dividing each ASV count by the total library size in order to yield their relative proportion of counts for each sample (Paulson et al., 2013).

Alpha diversity was studied with the R vegan package (Version: 2.5.6) using the Shannon and Simpson diversity indices. Differences between groups were assessed using Wilcoxon rank-sum tests or the exact Friedman rank sum with FDR correction in order to perform paired comparisons. Beta diversity was assessed through two distance matrices: (a) relative abundance with the Bray-Curtis index; and (b) presence/absence distance matrix using the binary Jaccard. Principal coordinate analysis (PCoA) was used to plot patterns of bacterial community diversity. The PERMANOVA analysis with 999 permutations was performed to reveal statistical differences.

### Analyzes of fatty acids (FAs) in the fecal samples

One hundred μl of a 1:10 dilution of feces (w/v) in phosphate buffer saline solution (PBS; pH 7.4) was supplemented with 100 μl of 2-ethyl butyric acid (Sigma-Aldrich, St. Louis, MO, USA) as an internal standard (1 mg/ml in methanol), and acidified with 100 μl of 20% formic acid (v/v). The acidic solution was then extracted with 1 ml of methanol and centrifuged for 10 min at 15,800 × *g*. Supernatants were kept at −20°C until analysis in a gas chromatography (GC) apparatus. The system used is composed of a 6,890 GC injection module (Agilent Technologies, CA, USA) with a HP-FFAP (30 m × 0.250 mm × 0.25 μm) column (Agilent Technologies) using a split/splitless injector in the split mode with a split ratio of 1:20. The injection volume of the samples was 1 μl. The injector and detector temperatures were kept at 240 °C and 250°C, respectively. The temperature of the column oven was set at 110°C, increased at 6°C/min to 170°C then increased at 25°C/min to 240°C, yielding a total GC run time of 18 min. Helium was used as carrier gas, at a constant flow rate of 1.3 ml/min. The chromatographic system was equipped with a flame ionization detector (FID). Data acquisition and processing were performed using ChemStation Agilent software (Agilent Technologies).

Statistical analyzes were performed using IBM SPSS Statistics v. 27.0.1 (IBM, Armonk, NY, USA). To examine the changes of the paired samples between the two time periods, we used the non-parametric Wilcoxon signed-rank test, and two-tailed probability values of *p* ≤ 0.05 were considered significant. In turn, medians, means, and interquartile ranges (IQR; Q1 and Q3) were represented in box and whisker graphics using Origin Pro-2021 software (OriginLab, Northampton, Massachusetts, USA).

## Results

### Evolution of the COVID-19 status of the participants

The mean age of the participants was, approximately, 85 years (Table 1). The recruited residents included 8 females and 7 males and all of them had several comorbidities and were polymedicated (Supplementary Table S1). A high percentage of them (*n* = 11; 73%) were SARS-CoV-2-positive at day 0 but, in contrast, all of them were negative at day 120. None of them became infected or re-infected with SARS-CoV-2 during the assay.

### Specific detection and quantification of *Ligilactobacillus salivarius* colonies and DNA in the fecal samples

The total *Lactobacillus* count oscillated between 5.7 and 7.7 log10 CFU/g at the beginning of the trial and increased slightly after the probiotic treatment (6.0–8.7 log10 CFU/g) (Table 2). In contrast, at the beginning of the trial, *L. salivarius* could not be detected in the feces of the participants. However, *L. salivarius* colonies were present in the samples of all the residents after the administration of the probiotic strain and its concentrations ranged between 4.4 and 7.1 log10 CFU/g (Table 2).

**Table 2:** Microbiological parameters, expressed as mean (95% CI), in the feces of the participants before (T1) and after 120 days of supplementation with *Ligilactobacillus salivarius* CECT 30632 (T2).

Parameter	T1	T2	*p*-value
**Colony-forming units (log_10_ CFU/g)**			
Total *Lactobacillus*	6.84 (6.53–7.15)	7.29 (6.97–7.62)	< 0.001
*L. salivarius*	nd	5.82 (5.38–6.26)	-
**qPCR (DNA copies/g)**			
*L. salivarius*	nd	6.42 (6.05–6.79)	-

nd, No detected in any sample.

Only those samples from which *L. salivarius* was cultured provided a positive result using the *L. salivarius*-specific qPCR assay (Table 2). Therefore, there was a complete qualitative agreement between both techniques (Table 2).

Finally, the *L. salivarius* isolates were genetically typified by the RAPD technique. Their profiles were identical to that of *L. salivarius* CECT 30632 (data not shown).

### Metataxonomic analysis of the nasal samples

The 16S rRNA gene sequencing analysis of the nasal samples yielded 653,484 high-quality filtered sequences, ranging from 17,098 to 44,506 per sample [median (IQR) = 29,686.5 (26,041.25– 33,718.5) sequences per sample].

**Figure 1:**
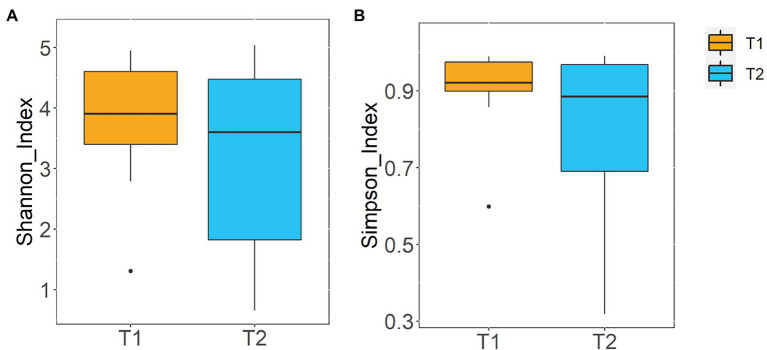
Alpha diversity at the ASV level of the nasal samples obtained at day 0 (T1) and at day 120 (T2). **(A)** Shannon diversity index; **(B)** Simpson diversity index.

Alpha diversity, as measured using the Shannon and Simpson diversity indices, was not significantly different when the nasal samples collected at day 0 [Shannon index = 3.90 (3.4–4.6); Simpson index = 0.92 (0.90–0.97)] were compared with those obtained at day 120 [Shannon index = 3.60 (1.82–4.48); Simpson index = 0.89 (0.69–0.97)] (*p* = 0.76 and *p* = 0.37, respectively) (Figure 1).

**Figure 2:**
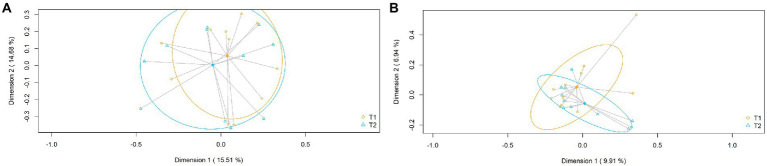
Beta diversity at the ASV level of the nasal samples obtained at day 0 (T1) and at day 120 (T2). **(A)** PCoA plots based on the Bray–Curtis dissimilarity index (relative abundance); **(B)** PCoA plots based on the Jaccard’s coefficient for binary data (presence of absence).

Beta diversity analysis at the ASV level revealed that nasal samples did not cluster according to the sampling times. When the nasal samples collected at day 0 were compared with those obtained at day 120, there were no statistical differences in relation to the relative abundance (Bray–Curtis distance matrix; *p* = 0.85) or to the presence/absence of different ASV sequences (binary Jaccard distance matrix; *p* = 0.70, respectively) (Figure 2).

Taxonomic analysis of the nasal sequences indicated that the bacterial profile was dominated by the phylum Firmicutes, followed by the phyla Proteobacteria, Bacteroidota, and Actinobacteriota. At the genera level, the 19 most abundant ones at both sampling times are shown in Table 3 and Supplementary Figure S1. However, no statistical changes were detected in the relative abundance of any of the main bacterial genera as a consequence of the administration of the probiotic strain.

**Table 3:** Relative frequencies, medians and interquartile range (IQR) of the relative abundance (%) of the most abundant bacterial phyla (in bold) and genera (in italics) detected in the nasal samples collected at day 0 (T1) and 120 (T2).

Phylum	T1		T2		*p*-value^2^
*Genus*	*n* (%)^1^	Median (IQR)	*n* (%)^1^	Median (IQR)	
**Firmicutes**	15 (100%)	61.43 (46.45–76.46)	15 (100%)	52.37 (42.61–67.50)	0.61
*Blautia*	15 (100%)	3.36 (1.13–5.26)	14 (93.33%)	1.27 (0.83–1.78)	0.12
*Subdoligranulum*	11 (73.33%)	3.06 (0.32–5.27)	12 (80%)	0.94 (0.12–3.73)	0.12
*[Eubacterium]_hallii_group*	14 (93.33%)	2.20 (0.98–6.13)	11 (73.33%)	0.68 (0.13–1.24)	0.01
*Christensenellaceae_R.7*	14 (93.33%)	1.10 (0.08–2.94)	13 (86.67%)	0.87 (0.09–4.64)	0.12
*Anaerostipes*	13 (86.67%)	2.00 (0.28–4.40)	13 (86.67%)	0.45 (0.16–1.42)	0.12
*Streptococcus*	15 (100%)	0.93 (0.4–1.95)	14 (93.33%)	0.54 (0.19–1.62)	0.30
*[Eubacterium]_coprostanoligenes_group*	14 (93.33%)	1.43 (1.09–2.16)	14 (93.33%)	1.66 (0.73–2.12)	1.00
*Clostridia_UCG.014*	9 (60.00%)	0.81 (<0.01–2.33)	10 (66.67%)	0.50 (<0.01–3.9)	0.30
*Oscillospiraceae-UCG.002*	13 (86.67%)	0.38 (0.18–1.81)	14 (93.33%)	1.46 (0.41–2.87)	0.30
*Faecalibacterium*	13 (86.67%)	0.60 (0.48–1.62)	15 (100%)	1.54 (1.02–2.73)	0.30
*Incertae_Sedis*	14 (93.33%)	0.60 (0.42–0.76)	14 (93.33%)	0.96 (0.27–1.42)	0.61
*Ruminococcus*	11 (73.33%)	0.57 (0.03–1.32)	12 (80%)	0.48 (0.17–2.70)	0.12
**Bacteroidota**	15 (100%)	13.64 (6.46–35.42)	15 (100%)	31.83 (20.72–38.89)	0.12
*Bacteroides*	15 (100%)	6.70 (1.41–24.92)	14 (93.33%)	14.67 (8.23–27.20)	0.12
*Alistipes*	15 (100%)	2.38 (0.48–3.36)	14 (93.33%)	2.48 (1.24–4.51)	0.61
*Parabacteroides*	15 (100%)	0.52 (0.21–2.66)	14 (93.33%)	3.34 (1.74–4.81)	0.12
**Proteobacteria**	15 (100%)	4.23 (1.26–7.44)	15 (100%)	5.61 (3.57–9.31)	0.61
*Escherichia/Shigella*	10 (66.67%)	0.06 (<0.01–3.47)	12 (80%)	1.45 (0.31–4.58)	0.12
**Actinobacteriota**	15 (100%)	4.01 (2.34–7.41)	15 (100%)	2.33 (0.80–2.86)	0.01
*Bifidobacterium*	14 (93.33%)	1.03 (0.80–5.43)	14 (93.33%)	0.36 (0.23–1.84)	0.30
**Verrucomicrobiota**	13 (86.67%)	2.81 (0.14–6.06)	14 (93.33%)	1.31 (0.36–3.47)	0.61
*Akkermansia*	11 (73.33%)	2.72 (0.11–5.80)	12 (80%)	1.18 (0.06–3.44)	0.61
Minor_phyla	15 (100%)	2.06 (0.69–4.45)	15 (100%)	2.66 (2.40–3.31)	0.30
Minor_genera	15 (100%)	30.26 (25.59–33.38)	15 (100%)	31.76 (25.06–41.04)	0.30
Unclassified_genera	15 (100%)	9.19 (7.77–20.95)	15 (100%)	9.95 (8.04–15.69)	0.61

1 *n* (%): Number of samples in which the phylum/genus was detected (relative frequency of detection).

2 Exact *p*-values for pairwise comparison of Friedman rank sum with FDR correction.

### Metataxonomic analysis of the fecal samples

The 16S rRNA gene sequencing analysis of the fecal samples yielded 844,567 high-quality filtered sequences, ranging from 21,950 to 35,737 per sample [median (IQR) = 28,144.5 (25,669.5 – 30,502.5) sequences per sample].

**Figure 3:**
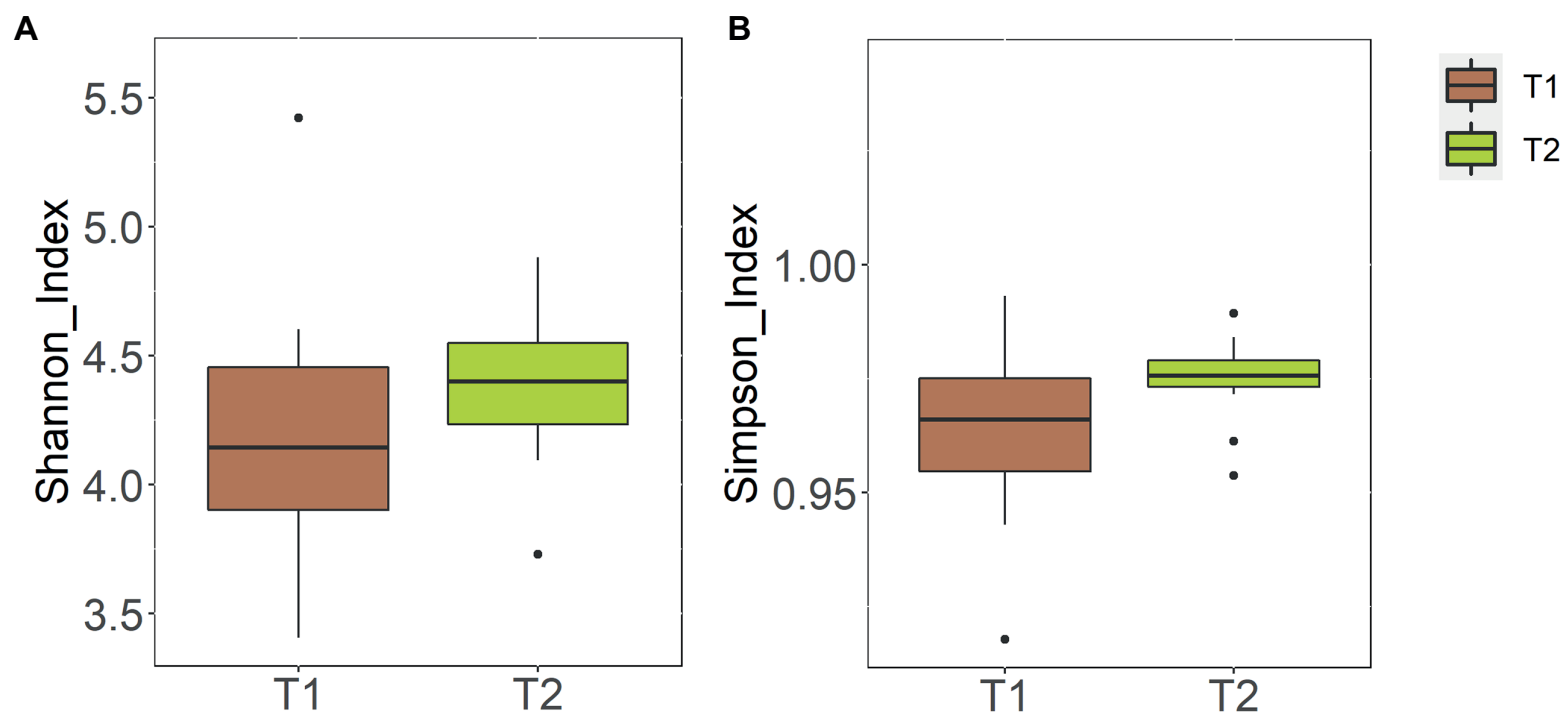
Alpha diversity at the ASV level of the fecal samples obtained at day 0 (T1) and at day 120 (T2). **(A)** Shannon diversity index; **(B)** Simpson diversity index.

**Figure 4:**
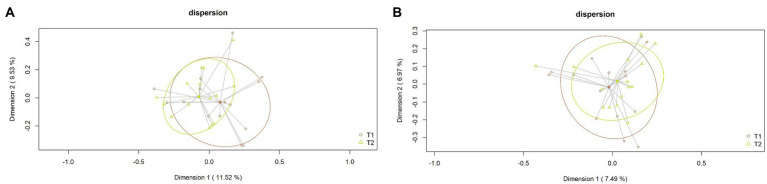
Beta diversity at the ASV level of the fecal samples obtained at day 0 (T1) and at day 120 (T2). **(A)** PCoA plots based on the Bray–Curtis dissimilarity index (relative abundance); **(B)** PCoA plots based on the Jaccard’s coefficient for binary data (presence of absence).

Again, alpha diversity was not significantly different between the samples collected at day 0 [Shannon index = 4.14 (3.90–4.46); Simpson index = 0.97 (0.95–0.98)] and those obtained at day 120 [Shannon index = 4.40 (4.23–4.55); Simpson index = 0.98 (0.97–0.98)] (*p* = 0.76 and *p* = 0.37, respectively) (Figure 3). Similarly, beta diversity analysis at the ASV level revealed that fecal samples did not cluster according to the sampling times (*p* = 0.51 in the case of the Bray–Curtis distance matrix and *p* = 0.82 in that of the binary Jaccard distance matrix) (Figure 4).

Taxonomic analysis of the fecal sequences indicated that the fecal bacterial profile was dominated by the phylum Firmicutes, followed by the phyla Bacteroidota, Proteobacteria, Actinobacteriota, and Verrucomicrobiota. At the genera level, the 19 most abundant ones at both sampling times are shown in Table 4; Supplementary Figure S2. Similarly to the nasal samples, no statistical changes were detected in the relative abundance of any of the main bacterial genera as a consequence of the administration of the probiotic strain.

**Table 4:** Relative frequencies, medians and interquartile range (IQR) of the relative abundance (%) of the most abundant bacterial phyla (in bold) and genera (in italics) detected in the fecal samples collected at day 0 (T1) and 120 (T2).

Phylum	T1		T2		*p*-value^2^
*Genus*	*n* (%)^1^	Median (IQR)	*n* (%)^1^	Median (IQR)	
**Firmicutes**	11 (100%)	48.83 (32.91–54.71)	11 (100%)	49.29 (13.33–64.27)	1.00
*Staphylococcus*	11 (100%)	15.37 (4.08–36.40)	11 (100%)	5.05 (0.48–35.83)	0.55
*Streptococcus*	11 (100%)	1.16 (0.74–4.60)	9 (81.82%)	0.32 (0.08–1.10)	0.07
*Anaerococcus*	8 (72.73%)	0.94 (0.13–4.30)	7 (63.64%)	0.18 (<0.01–3.37)	0.55
*Peptoniphilus*	8 (72.73%)	0.64 (0.05–2.56)	6 (54.55%)	0.19 (<0.01–5.60)	1.00
*Dolosigranulum*	6 (54.55%)	0.03 (<0.01–1.70)	6 (54.55%)	0.04 (<0.01–0.53)	1.00
*Anoxybacillus*	10 (90.91%)	1.77 (0.65–3.05)	10 (90.91%)	1.67 (0.28–2.58)	0.23
*Finegoldia*	7 (63.64%)	0.29 (<0.01–1.58)	4 (36.36%)	<0.01 (<0.01–1.46)	1.00
**Proteobacteria**	11 (100%)	20.28 (10.92–27.91)	11 (100%)	20.78 (15.74–41.52)	0.55
*Pseudomonas*	5 (45.45%)	<0.01 (<0.01–0.12)	4 (36.36%)	<0.01 (<0.01–0.13)	1.00
*Serratia*	2 (18.18%)	<0.01 (<0.01–<0.01)	3 (27.27%)	<0.01 (<0.01–0.10)	1.00
*Moraxella*	1 (9.09%)	<0.01 (<0.01–<0.01)	1 (9.09%)	<0.01 (<0.01–0.13)	1.00
*Colwellia*	10 (90.91%)	2.06 (1.05–4.10)	11 (100%)	1.90 (0.14–3.04)	0.55
*Sulfitobacter*	9 (81.82%)	0.65 (0.32–1.6)	8 (72.73%)	0.41 (0.02–1.50)	1.00
*Litoreibacter*	9 (81.82%)	0.94 (0.51–1.05)	7 (63.64%)	0.35 (<0.01–0.75)	0.23
*Sphingorhabdus*	9 (81.82%)	0.78 (0.25–1.47)	10 (90.91%)	0.35 (0.09–0.76)	0.23
**Bacteroidota**	11 (100%)	10.89 (5.06–14.28)	11 (100%)	9.17 (1.00–13.13)	1.00
*Maribacter*	10 (90.91%)	1.56 (0.68–2.46)	11 (100%)	1.95 (0.35–2.46)	1.00
*Prevotella*	7 (63.64%)	0.06 (<0.01–0.54)	5 (45.45%)	<0.01 (<0.01–0.23)	1.00
**Actinobacteriota**	11 (100%)	7.18 (4.17–19.46)	11 (100%)	4.27 (2.27–7.20)	0.23
*Corynebacterium*	10 (90.91%)	4.50 (0.31–11.91)	10 (90.91%)	1.57 (0.43–2.01)	0.23
Cyanobacteria	10 (90.91%)	0.46 (0.29–0.68)	10 (90.91%)	0.51 (0.16–1.01)	1.00
Chloroplast	10 (90.91%)	0.46 (0.29–0.68)	9 (81.82%)	0.48 (0.16–0.98)	1.00
Minor_phyla	11 (100%)	6.81 (4.67–8.18)	11 (100%)	5.44 (0.49–8.47)	0.23
Minor_genera	11 (100%)	21.17 (12.70–27.54)	11 (100%)	12.69 (1.56–29.85)	0.55
Unclassified_genera	11 (100%)	10.7 (5.33–17.43)	11 (100%)	7.96 (0.90–13.82)	0.55

1 *n* (%): Number of samples in which the phylum/genus was detected (relative frequency of detection).

2 Exact *p*-values for pairwise comparison of Friedman rank sum with FDR correction.

### Profiles of fatty acids in the fecal samples

There were no statistically significant differences in the levels of total fatty acids (FAs) between the two sampling times (*p* = 0.925). Similarly, there were no statistically significant differences in the fecal concentration of the three main short chain fatty acids (SFCAs) between the two sampling times (acetic: *p* = 0.551; propionic: *p* = 0.972; butyric: *p* = 0.646) (Table 5). In addition, the sum of the concentrations of these three main SCFAs was similar at both time points (*p* = 0.875) (Table 5) and, also, the acetic/propionic ratio (*p* = 0.382).

**Table 5:** Concentration (μg/g) and frequency of detection (% samples) of fecal fatty acids (FAs) at day 0 (T1) and 120 (T2). The *p*-values are related to the concentration de the FAs at both sampling times.

Fatty acid	T1		T2		*p*-values
	Concentration	% samples	Concentration	% samples	
**SCFAs**					
Acetic	3272.01 ± 2760.79	100	3100.50 ± 1848.35	100	0.551
Propionic	1146.10 ± 926.92	93	946.31 ± 669.98	100	0.972
Isobutyric	249.60 ± 148.62	57	173.06 ± 160.12	57	
Butyric	851.30 ± 1169.79	93	576.03 ± 733.90	71	0.646
Acetic/propionic ratio	3.19 ± 1.04		3.96 ± 1.57		0.382
Total	5126.74 ± 4715.54	100	4458.26 ± 3043.43	100	0.875
**BCFAs**	647.80 ± 525.95	93	459.92 ± 478.57	100	
Isovaleric	494.20 ± 360.17	93	388.80 ± 329.07	93	0.388
Isobutyric	249.60 ± 148.62	57	173.06 ± 160.12	57	0.173
**MCFAs**	283.67 ± 313.72		111.02 ± 121.66		
Caproic	283.67 ± 313.72	21	111.02 ± 121.66	36	0.593
**Total FAs**	6022.21 ± 5462.42	100	5101.11 ± 3705.72	100	0.925

In relation to branched chain fatty acids (BCFAs), formed by isobutyric and isovaleric acids, their concentrations were similar in the two sampling times evaluated in this study (*p* = 0.173 and *p* = 0.388 for isobutyric and isovaleric acid, respectively) (Table 5).

The number of samples in which caproic acid, a medium-chain fatty acid (MFCA), was detectable was particularly low (*n* ≤ 5 in each sampling point), and although its concentration tended to decrease after the administration of the probiotic strain (Table 5), the difference did not reach a statistically significant value (*p* = 0.593).

## Discussion

The elderly was the most affected population during the first months of the COVID-19 pandemic. Within this population, those living in nursing homes constituted a particularly vulnerable group, characterized by high rates of infection and death (Lai et al., 2020; Araújo et al., 2021). These high mortality rates have been linked to the accumulation of risk factors or comorbidities that are typically associated with aging (Trecarichi et al., 2020; Heras et al., 2021; Suñer et al., 2021), together with high levels of community and intra-home transmission and the lack of proper policy responses in relation to the situation in nursing homes (Sepulveda et al., 2020). The situation was particularly worrying in Spain since this country has one of the world’s highest aging index while the percentage of elderly living in nursing homes is also high (Aguilar-Palacio et al., 2022).

The nursing home that we selected for the trial was severely affected by COVID-19. Immediately before the pandemic, there were 47 older people living in this care center but it has a devastating impact on the residents when it reached the village. In a few weeks, approximately 40% (*n* = 18) of them died (10 with acute COVID-19-related symptoms) although none was tested for SARS-CoV-2. Later, the 29 surviving residents and the workers were PCR tested and most of them (> 80% of the residents and all the workers except one) were PCR-positive.

Initially, we investigated the effect of *L. salivarius* CECT 30632 on the functional (Barthel index), cognitive (GDS/FAST) and nutritional (MNA) status, and on the nasal and fecal inflammatory profiles of the 25 recruited participants that fulfilled the inclusion criteria (Mozota et al., 2021). After the trial, no changes in the cognitive score were detected but the cognitive and nutritional status improved significantly. In addition, the concentrations of some immune factors changed significantly after the consumption of the probiotic strain. Among them, it must be highlighted that the concentrations of some immune factors used as biomarkers of acute viral respiratory infections, such as BAFF/TNFSF13B, APRIL/TNFSF13, or IL-8 (Alba et al., 2021), decreased significantly (Mozota et al., 2021). Since we kept aliquots of the samples obtained at both sampling times from a subset of 15 residents, in this subsequent study we evaluated the effect of the administration of the strain on the nasal and fecal microbiota and in the fecal fatty acid profile of the recruited people.

Culture-based methods showed that *L. salivarius* (species level) could not be detected in any of the fecal samples at day 0. It has already been described that aging is associated with the absence of *L. salivarius* from the gut microbiome (Le Roy et al., 2015). In contrast, *L. salivarius* CECT 30632 (strain level) was present in all the recruited people at day 120 at concentrations ranging from 4.4 to 7.1 log10 CFU/g. Since we did not identify and genotype all the colonies growing on MRS-Cys plates, we cannot completely discard that other *L. salivarius* strains may be also present in the fecal samples of the participants. However, the lack of isolation and PCR detection of *L. salivarius* at baseline makes likely the possibility that all the *L. salivarius*-like colonies belonged to the administered strain. These values indicates that the strain was able to survive the transit through the digestive tract and to reach the gut in relatively high concentrations. It must be taken into account that the actual gut concentrations might be substantially higher since we only tested fecal samples and, therefore, the concentration of the strain attached to the gut mucosa remains unknown. The gut microbiota is an ecological succession (Falony et al., 2018), where the abundance of bacteria changes across the intestine, meaning that a particular strain could be highly abundant in a proximal segment of the intestine and less abundant in a distal segment, hence leading to lower counts in stools. Culture-based methods and *L. salivarius*-specific qPCR evidenced that, at least, there was a change affecting to one species (*L. salivarius*) and one strain (*L. salivarius* CECT 30632) in the microbiota of the recruited individuals.

Paradoxically, the increase in the *L. salivarius* counts was not reflected in changes in the metataxonomic analysis of the fecal samples. This fact may be due to the fact that the metataxonomic approach used in this study (targeting the V3-V4 hypervariable regions of the 16S rRNA gene) does not allow a proper discrimination at the species level and, as a consequence, we could not assess the change in the relative abundance of the sequences corresponding to the *L. salivarius* species. So far, most of the metataxonomic analysis performed to study the human bacteriome have relied in methods which only allow a proper discrimination at the taxonomic level of genus or higher. In order to achieve species or strain level discrimination, it will be necessary to apply developing procedures, including full-length 16S rRNA gene sequencing (Yang et al., 2020; Zhang et al., 2021) and metagenome-assembled genomes (Arikawa et al., 2021; Holman et al., 2022), and/or the combination of culture-dependent and independent techniques.

The metataxomonic analysis did not find differences between both sampling times in relation to the relative abundance of the genus *Lactobacillus* (*sensu* lato). This is not surprising since the probiotic treatment led to a very moderate increase in the total *Lactobacillus* counts. Although studies addressing the impact of aging on the fecal levels of *Lactobacillus* have provided contradictory results (Salazar et al., 2020), this genus is not among the most abundant ones in feces of elderly people (Claesson et al., 2011, 2012; Albrich et al., 2022; Yan et al., 2022). In addition, hypertension, which is a very common comorbidity among the elderly and a risk factor for COVID-19, has been linked to a depletion in *Lactobacillus* levels (Ghosh et al., 2020). In this study, most of the recruited elderly presented this comorbidity (Supplementary Table S1). Therefore, it is highly probable that small increases in the percentage of sequences of a low abundance genus may remain undetected using a metataxonomic approach based on partial 16S rRNA gene sequences. The number of sequences per sample obtained in this work (from 21,950 to 35,737) allows the detection of shifts related to the most abundant genera but it may be not enough to detect shifts in the populations of the rarest genera, which relative abundance may be 10,000 to 100,000 times lower. This highlights the importance of a suitable sequencing depth for being able to detect changes affecting low abundance genera or species. The discrepancy between culture-based methods, qPCR and 16S rRNA sequencing observed in this study reveals that metataxonomic approaches may be unsuited to detect changes readily measurable by culture-based methods or qPCR. This fact is in agreement with a recent study which demonstrated that low abundance bacteria can train immunity (Han et al., 2022).

The administration of the probiotic strain did not change the fatty acid profiles in the fecal samples of the recruited people, a finding that is in agreement with the lack of metataxomic changes observed in this study. Interestingly, it has been reported that there were no differences in fecal SCFA concentrations among people with different *Lactobacillus* counts (Le Roy et al., 2015). Since *Lactobacillus* sp. are not the main SCFAs producers and many other bacterial groups are able to produce higher SCFAs amounts as a result of gut fermentation processes, the moderate increase in total *Lactobacillus* observed in the recruited elderly may have not been enough to increase the fecal SCFAs values in their fecal waters, either by the probiotic strain itself or by fostering metabolic cross-feeding interactions with other SCFAs-producing bacteria.

SCFAs are a result of the metabolism of the gut microbiota and play several beneficial roles for the host health (Puertollano et al., 2014; Morrison and Preston, 2016; van der Beek et al., 2017). Aging-related disturbances in the composition of the gut microbiota are typically associated with lower levels of SCFAs and an enrichment in the pathways responsible for the degradation of SCFAs (Yan et al., 2022). Although it has been observed that patients with COVID-19 had an impaired production of SCFAs by their gut microbiomes (Zhang et al., 2022), these metabolites seem unable to prevent the entry and replication of SARS-CoV-2 in gut cells (Pascoal et al., 2021).

Overall, the results of this work and those obtained in a previous study with the same cohort indicate that the strain was able to reach the gut in all the recruited elderly, and suggest that it was able induce beneficial immune responses both at the respiratory and gut level, contributing to an improvement in functional and nutritional scores (Mozota et al., 2021). COVID-19 is associated with the overproduction of proinflammatory cytokines (Zazzara et al., 2022); in this pandemic context, immune enhancement functions are particularly relevant for the elderly, because of the immunosenescence associated with aging (Aw et al., 2007; Wang et al., 2022), but, most especially, for those residing in nursing homes. A study assessing microbiota–health correlations among elderly found that the serum levels of several markers of inflammation (TNF-α, IL-6 and IL-8, and C-reactive protein) were significantly higher among subjects living in long-stay nursing homes than among community dwellers (Claesson et al., 2012). Long-stay elderly also obtained poorer scores for comorbidity, functionality, nutritional state, muscle mass, and mental activity (Claesson et al., 2012).

The association of elderly with inflammation argues in favor of approaches enabling immunomodulation (Guigoz et al., 2008), such as the use of probiotics. This was particularly challenging in the frame of the COVID-19 pandemic. The first published studies about the use of probiotics in hospitalized COVID-19 patients described a positive effect, including a reduction in the duration of diarrheal episodes, in the risk of respiratory failure and/or in the risk of death (d’Ettorre et al., 2020; Ceccarelli et al., 2021). However, such studies did not address the potential mechanisms responsible for the observed benefits. More recently, it was reported that a *Lactiplantibacillus plantarum* strain was able to induce innate cytokine responses with the potential for providing a protection against the more severe courses of this disease (Kageyama et al., 2022). Similarly to our results, other studies involving the oral administration of a probiotic formula to COVID-19 outpatients did not find significant changes in the composition of the fecal bacteriome as a result of the probiotic intake (Gutiérrez-Castrellón et al., 2022). The authors suggested that the probiotic product primarily acted by interacting with the host immune system since they observed increased titers of anti-SARS-CoV2 specific antibodies compared to placebo.

This study faces some limitations. Because of the low number of participants, the lack of randomization, and the lack of a placebo group, the observed beneficial effects must be confirmed in future well-designed placebo-controlled trials involving a high number of elderly. However, our results indicate that *L. salivarius* CECT 30632 colonized, at least temporarily, the intestinal tract of the recruited elderly and may have contributed to improvements in their functional, nutritional, and immunological status, without changing the general structure of their nasal and fecal bacteriomes when assessed at the genus level.

## Data availability statement

The datasets presented in this study can be found in online repositories. The names of the repository/repositories and accession number(s) can be found at: https://www.ncbi.nlm.nih.gov/bioproject/PRJNA880542/.

## Ethics statement

The studies involving human participants were reviewed and approved by Ethics Committee of the Hospital Clínico San Carlos (Madrid, Spain; protocol: CEIC 20/263-E_COVID; date of approval: 01/04/2020, act 4.1/20). The patients/participants provided their written informed consent to participate in this study.

## Author contributions

JR: conceptualization. MM, IC, NG-T, RA, SD, and IG-D: methodology. CA: software. JR and SD: resources and writing—review and editing, funding acquisition. NG-T, CA, SD, and JR: data curation. JR, CA, and SD: writing—original draft preparation. All authors have read and agreed to the published version of the manuscript.

## Conflict of interest

The authors declare that the research was conducted in the absence of any commercial or financial relationships that could be construed as a potential conflict of interest.

## Publisher’s note

All claims expressed in this article are solely those of the authors and do not necessarily represent those of their affiliated organizations, or those of the publisher, the editors and the reviewers. Any product that may be evaluated in this article, or claim that may be made by its manufacturer, is not guaranteed or endorsed by the publisher.

## Footnotes

^1^ https://www.R-project.org
