# Supplementary figures and images for: Administration of Ligilactobacillus salivarius CECT 30632 to elderly during the COVID-19 pandemic: Nasal and fecal metataxonomic analysis and fatty acid profiling
Source: Front Microbiol. 2022 Dec 16;13:1052675. doi: 10.3389/fmicb.2022.1052675 (PMC9800801; doi:10.3389/fmicb.2022.1052675)

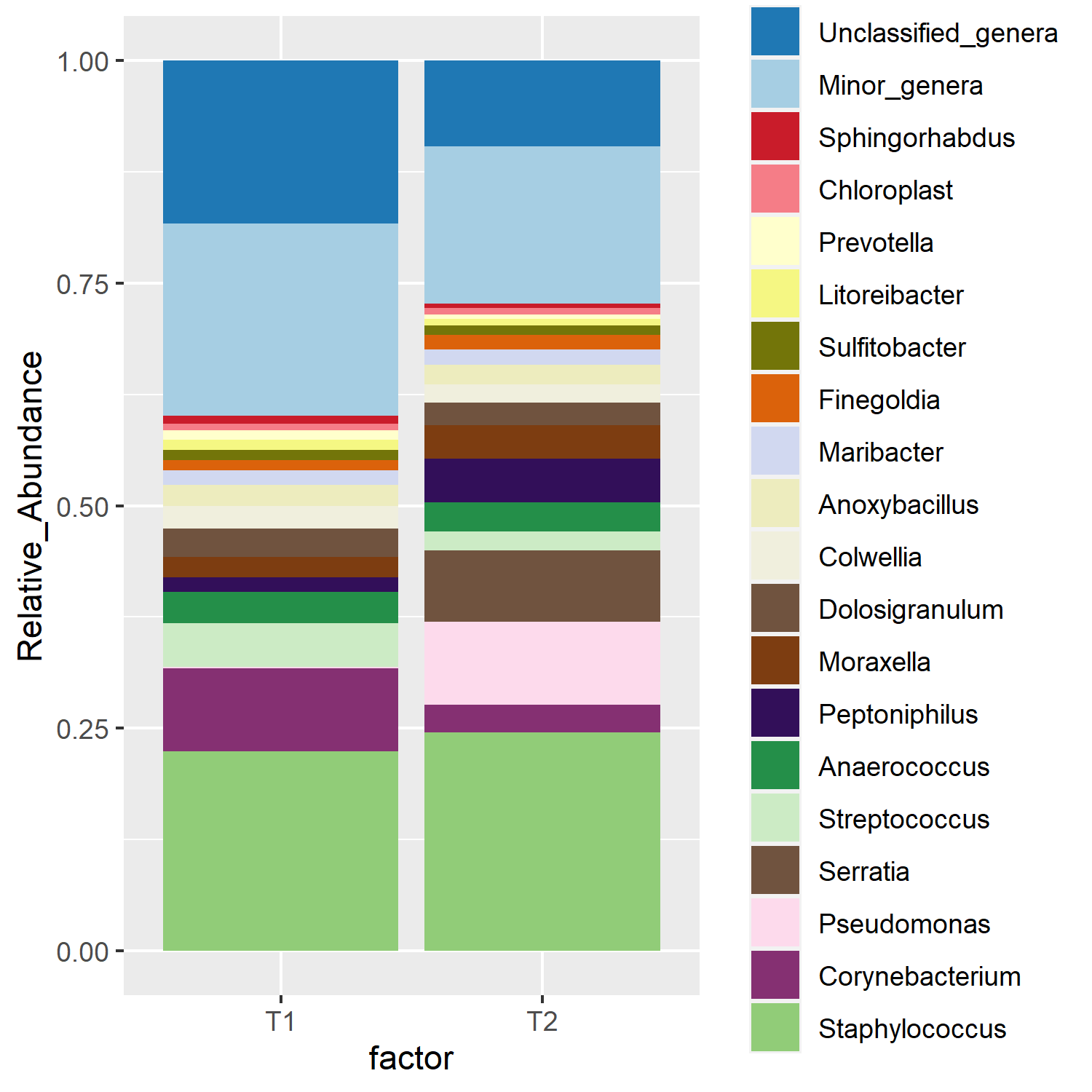

Supplement: Supplementary file 1 [file Image_1.TIFF]

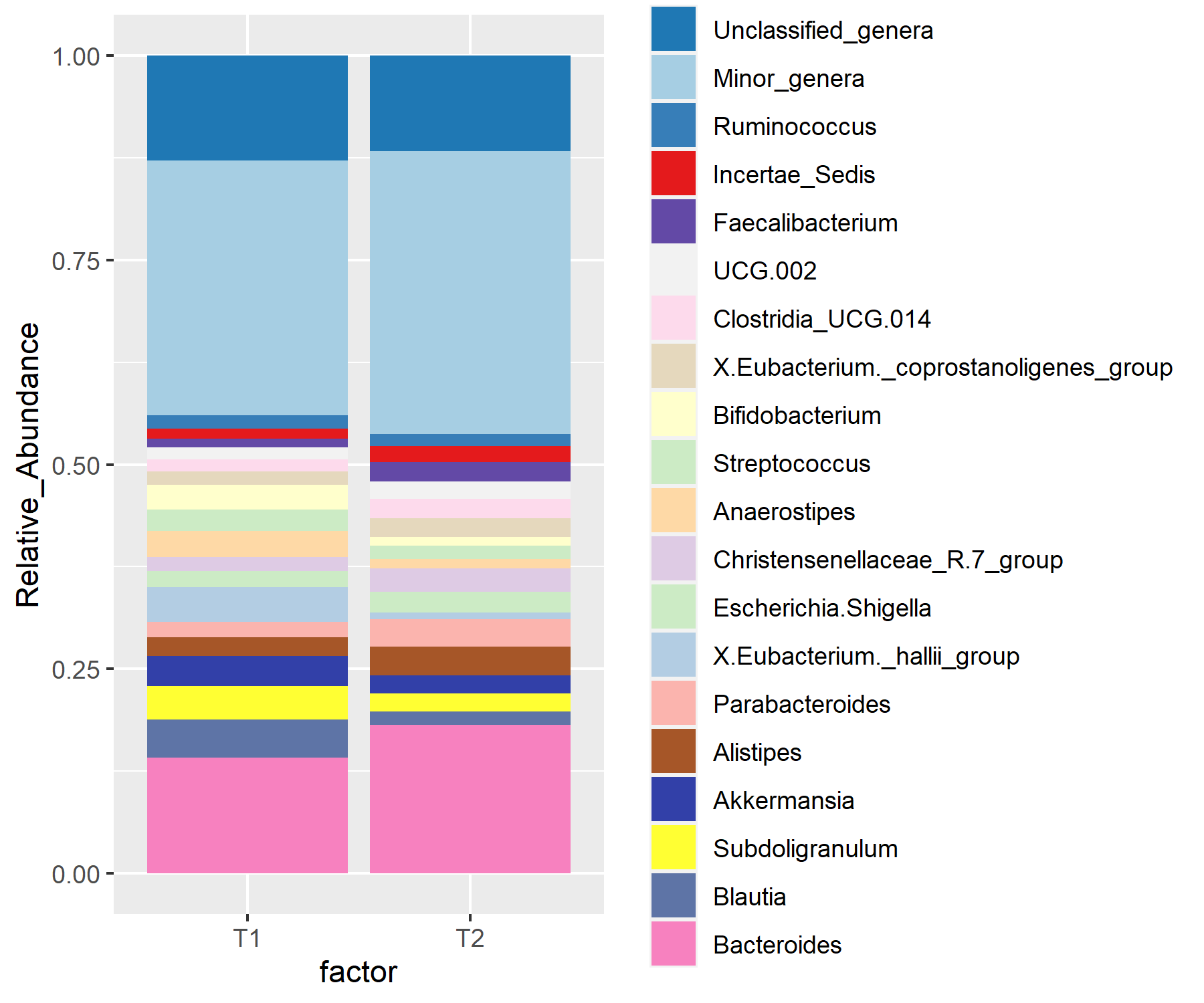

Supplement: Supplementary file 2 [file Image_2.TIFF]
